# A Current Sensor Based on the Giant Magnetoresistance Effect: Design and Potential Smart Grid Applications

**DOI:** 10.3390/s121115520

**Published:** 2012-11-09

**Authors:** Yong Ouyang, Jinliang He, Jun Hu, Shan X. Wang

**Affiliations:** 1 State Key Lab of Power Systems, Department of Electrical Engineering, Tsinghua University, Beijing 100084, China; E-Mails: ouyy07@gmail.com (Y.O.Y.); hjun@tsinghua.edu.cn (J.H.); 2 Center for Magnetic Nanotechnology, Stanford University, 450 Serra Mall, Stanford, CA 94305, USA; E-Mail: sxwang@stanford.edu

**Keywords:** current sensing, giant magnetoresistance, sensor design, smart grid

## Abstract

Advanced sensing and measurement techniques are key technologies to realize a smart grid. The giant magnetoresistance (GMR) effect has revolutionized the fields of data storage and magnetic measurement. In this work, a design of a GMR current sensor based on a commercial analog GMR chip for applications in a smart grid is presented and discussed. Static, dynamic and thermal properties of the sensor were characterized. The characterizations showed that in the operation range from 0 to ±5 A, the sensor had a sensitivity of 28 mV·A^−1^, linearity of 99.97%, maximum deviation of 2.717%, frequency response of −1.5 dB at 10 kHz current measurement, and maximum change of the amplitude response of 0.0335%·°C^−1^ with thermal compensation. In the distributed real-time measurement and monitoring of a smart grid system, the GMR current sensor shows excellent performance and is cost effective, making it suitable for applications such as steady-state and transient-state monitoring. With the advantages of having a high sensitivity, high linearity, small volume, low cost, and simple structure, the GMR current sensor is promising for the measurement and monitoring of smart grids.

## 1. Introduction

Smart grids are the latest trend in the World's power system. They represent an evolution towards a more optimized and sustainable energy system using the implementation of information technology [1–3]. Advanced sensing and measurement techniques are key technologies in a smart grid to successfully provide accurate information for real-time monitoring and control of the entire distributed power system.

The giant magnetoresistance (GMR) effect was discovered in some ferromagnetic metallic materials. The resistance of these materials decreases greatly under the influence of external magnetic field. Grünberg and Fert separately discovered this phenomenon in 1988 [4–6], which revolutionized the field of data storage and magnetic measurement. For their significant contributions, they were awarded the 2007 Nobel Prize for Physics [7]. Since IBM Corporation developed the first commercial read head based on the GMR effect in 1997, the area density of the devices has grown hundreds of times [8,9].

In the field of magnetic measurement, GMR sensors are widely utilized for position and angular sensing, current sensing and biological sensing. A number of integrated detectors containing GMR elements with wide bandwidth have been developed for motor drives, power electronic modules, and regulator modules [9–13]. A GMR sensor for highly-sensitive stress measurement has been introduced [14]; additionally, an eddy current testing probe based on the spin-valve GMR effect has been developed for inspecting printed circuit boards [15]. In the field of biological sensing, GMR sensors have been applied in crop production for sensing growth conditions, fertilization, and irrigation; GMR sensors are also useful for plant protection, harvesting, and fleet controls [16]. Sensors based on the GMR effect have also been used in protein and DNA assays, to perform molecular diagnostics of diseases [17–20].

In order to realize linear sensing, the sensed field range, linearity, and sensitivity are three important parameters. For example, the sensitivity is 25 mV·A^−1^, and the sensed field range is from 0 to ±10 mA in certain linear spin-valve GMR sensors reported in the literature [21–24].

In this work, we designed the structure of a GMR sensor for current sensing that can be implemented in a smart grid. In addition, the characteristics of the GMR sensor, such as its static characteristics, dynamic characteristics and thermal characteristics were tested.

## 2. Principle of Operation

**Figure 1.**
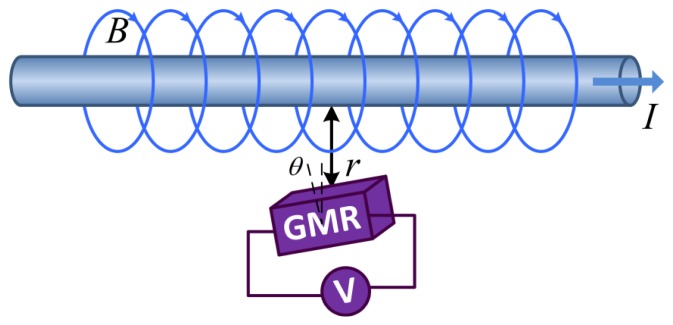
Principle of operation of the GMR current sensor. *I* is the current passing through the wire, and the direction is from left to right. *B* is the magnetic field generated by the current *I*, and the direction is rotating around the wire. *r* is the distance from the wire to the sensor, and *θ* is the angle between the magnetic field direction and the axis of sensitivity of the GMR chip.

Current flowing through a metal wire creates a magnetic field around the wire. The relationship between the current and magnetic field is described by the Biot-Savart Law. The principle of operation of the GMR current sensor is shown in Figure 1. The current flowing through the metal wire can be measured from the output of the GMR current sensor, when the GMR current sensor is placed close to the wire. The output of the GMR current sensor can be generally described by:

(1) $$V=F_{\text{GMR}}(I,f,T,V_{S},\mu ,r,\theta ,B_{D})$$

where *V* is the output voltage of the GMR current sensor, *I* and *f* are the magnitude and frequency of the current passing through the wire, respectively, *T* is the environmental temperature, *V_S_* is the power supply voltage of the sensor, *μ* is the relative permeability around the sensor, *r* is the distance from the wire to the sensor, *θ* is the angle between the magnetic field direction and the axis of sensitivity of the GMR chip, and *B_D_* is the surrounding parasitic magnetic field.

## 3. Sensor Design

**Figure 2.**
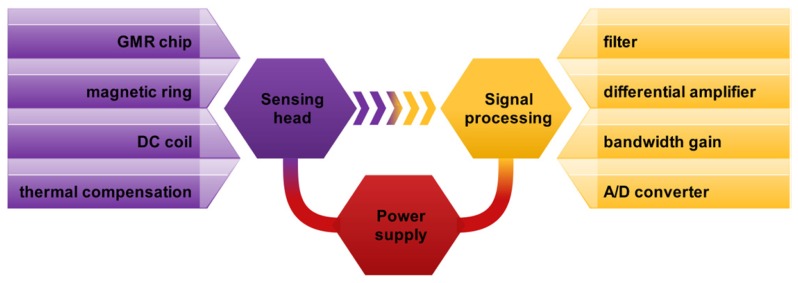
Block diagram of sensor system. The whole sensor system contains three main subsystems: The function of the sensing head is to convert the current signal measured to the voltage signal. The function of the signal processing subsystem is to process the voltage signal from the sensing head and calculate the current measured. The function of the power supply subsystem is to provide the power source for the whole sensor system.

The sensor system contains three main subsystems: the sensing head, the signal processing subsystem and the power supply subsystem. Each subsystem affects the overall performance of the sensor. The block diagram of the sensor system is shown in Figure 2.

### 3.1. Sensing Head

**Figure 3.**
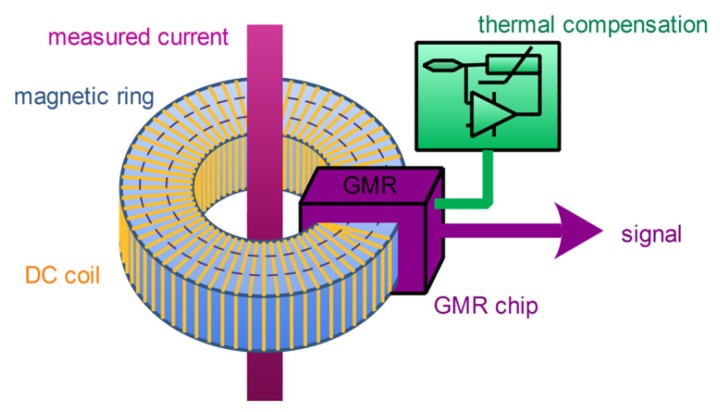
Schematic diagram of sensing head.

The sensing head, which is shown in Figure 3, is the kernel of the whole measuring system, and its function is to convert the current signal measured into the voltage signal that can be measured easily. A wire with the current line to be measured passes through a magnetic ring. A DC coil is uniformly wrapped around the magnetic ring. A GMR chip is placed in the gap of the magnetic ring, and a thermal compensation unit is used to compensate the temperature drift of the GMR chip. The output of the GMR chip is subsequently processed by the signal processing subsystem.

**Figure 4.**
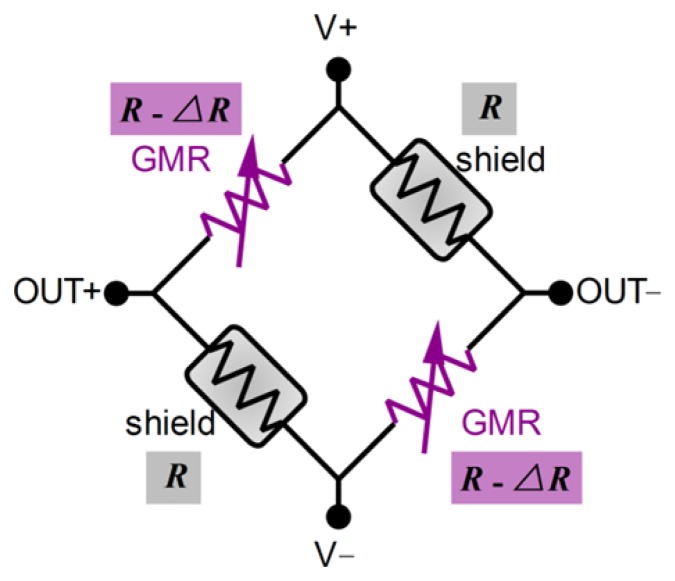
Wheatstone bridge of GMR chip. The two shield resistors are shielded elements, and the two GMR resistors are active elements. V+ and V− are two terminals of the power supply of the GMR chip. OUT+ and OUT− are the positive and negative output of the GMR chip.

In order to convert the magnetic signal to a voltage signal that can be easily measured, a Wheatstone bridge is used in the design of the GMR chip (Figure 4). In the GMR chip, four resistive elements are arranged as follows: two are active sensing elements and two are shielded elements. Due to limitations in material processing and manufacturing technology, the four resistive elements cannot be manufactured identically, so their magnitudes are slightly different when the magnetic field is not present.

The output of the GMR chip is differential, and can be described as follows:

(2) $$\begin{array}{c} {\text{Vout}}_{+}=\frac{R}{2R-\Delta R}({\text{V}}_{+}-{\text{V}}_{-})+{\text{V}}_{-}\\ {\text{Vout}}_{-}=\frac{R-\Delta R}{2R-\Delta R}({\text{V}}_{+}-{\text{V}}_{-})+{\text{V}}_{-}\\ {\text{Vout}}_{+}-{\text{Vout}}_{-}=\frac{\Delta R}{2R-\Delta R}({\text{V}}_{+}-{\text{V}}_{-})\approx \frac{\Delta R}{2R}({\text{V}}_{+}-{\text{V}}_{-}) \end{array}$$

where *Vout_+_* and *Vout*_−_ are the voltage outputs of the GMR chip, *V_+_* and *V*_−_ are the power supply voltages of the GMR chip, and *R* is the intrinsic resistance of the four elements when no magnetic field is applied. Δ*R* is the change of the resistance of the two active elements under a magnetic field, and Δ*R* is linearly proportional to the magnetic field to be measured.

The MR ratio (Δ*R/R*) is about 7%. Because Δ*R* is much smaller than *R*, the output of the GMR chip can be treated as linearly proportional to Δ*R*.

**Figure 5.**
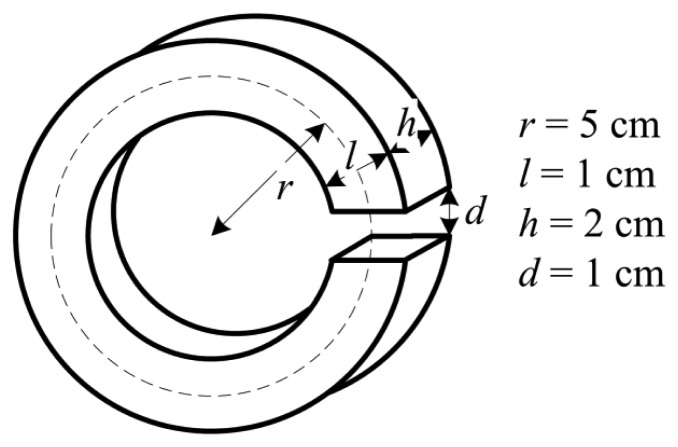
The structure of the magnetic ring. The magnetic ring is made of silicon steel. The size of the magnetic ring is as follows: the average radius *r* is 5 cm, the width *l* is 1 cm, the thickness *h* is 2 cm, and the gap *d* is 1 cm.

The magnetic ring is made of a soft ferromagnetic material with a large relative permeability, and its structure is shown in Figure 5. It can make the magnetic field to remain stable and nearly unaffected by position fluctuation of the current line. The magnetic field in the gap of the magnetic ring can be described as:

(3) $$B=\frac{\mu_{0}I}{d+\frac{2\pi r-d}{\mu_{r}}}\approx \frac{\mu_{0}I}{d}$$

where *B* is the flux density, *μ*_0_ is the permeability of vacuum, *μ_r_* is the relative permeability of the magnetic ring, *r* is the average radius of the magnetic ring, *d* is the length of the gap, and *I* is the measured current.

Because *μ_r_* of ferromagnetic materials is usually in the range of 1,000 to 10,000, then (2*πr*–*d*)/*μ_r_* ≈ 0. Therefore, *B* approximately only depends on *d* and *I*. Moreover, the magnetic ring can greatly amplify the magnetic field to be measured, enabling the GMR current sensor to measure a smaller current. The magnetic ring can also shield unwanted signals, such as neighboring current lines and the geomagnetic field.

The number of DC coil turns wound around the magnetic ring is 500, and the current of DC coil is 10 mA. Therefore, the DC magnetic field in the gap is about 6.28 Oe.

The GMR chip used in the experiment is a product of the NVE Corporation (Part Number: AA002-02); and its multilayered structure is based on a Ni-Fe-Co magnetic layer and Cu spacer. The typical transfer curve of the multilayered GMR chip is inverse-V-shaped. As it cannot identify the direction of the magnetic field to be measured; the DC coil superposes the magnetic field created by the passing current; so the GMR chip works on unipolar field; which can reduce hysteresis and realize bipolar measurement. Under a constant voltage supply, the GMR sensor signal output will fall as temperature rises. Because the GMR sensor signal is related to the power supply, a positive temperature control voltage supply is added to feed the GMR current sensor, as shown in Figure 3, to compensate for the temperature drift.

### 3.2. Signal Processing

**Figure 6.**
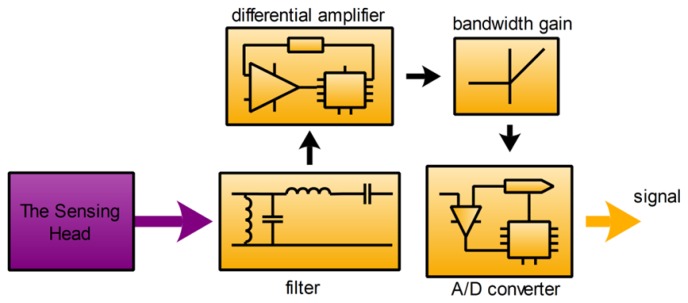
Schematic diagram of signal processing subsystem.

The signal processing subsystem is used to process the voltage signal from the sensing head and calculate the current measured, and its schematic is shown in Figure 6.

As shown in the Figure 6, the filter is a low-pass filter used to remove some interfering signals, especially high frequency noise of the voltage signal from the sensing head. The output of the GMR chip includes common-mode signal, and it is about half of the power supply. The differential amplifier is used to remove useless common-mode noise, and to amplify useful differential signal, and the model used in the experiment is an instrumentation amplifier produced by Texas Instruments (Part Number: INA114). The bandwidth gain unit is used to improve the frequency response of the GMR current sensor. Finally, the A/D converter is used to convert the analog signal from the GMR chip to digital signal, and the model used in the experiment is an USB digitizer produced by National Instruments (Part Number: USB-5133).

### 3.3. Power Supply

The power supply subsystem is used to provide a stable power source for the whole system, such as the voltage source of the GMR chip, the adjustable current source of the DC coil, and reference voltages of the system. A MH-Ni rechargeable battery pack is applied to the power source of the system. Because the voltage of battery pack fluctuates from 13 V to 17 V, voltage regulators are used to provide stable voltages for the whole system.

### 3.4. Circuit Design

**Figure 7.**
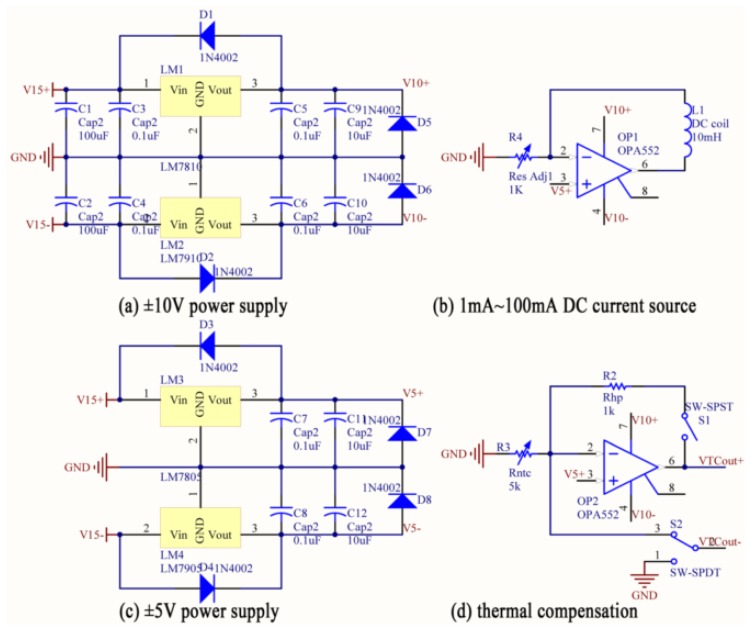

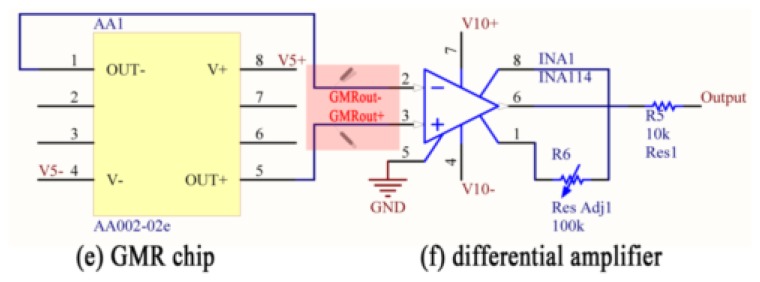
Circuit diagram of the sensor system. The circuit diagram contains the power supply subsystem (**a**, **b**, **c**), the sensing head subsystem (**d**, **e**) and the differential amplifier unit (**f**). VTCout+ and VTCout- are outputs of the thermal compensation unit, connected to power supply to the GMR chip. Output is the output of the differential amplifier, and is connected to the USB digitizer (USB-5133). GMROut+ and GMROut-, two red measuring probes shown in the figure, are outputs of the GMR chip. In order to get the typical performance of the GMR current sensor and eliminate the influence of the thermal compensation unit and the differential amplifier unit, the power supply of the GMR chip is ±5 V, and the direct measuring terminals (GMROut+ and GMROut-) are the outputs of the GMR chip in the experiment.

The circuit diagram of the sensor system is shown in Figure 7. It contains three parts: the power supply subsystem, the sensing head and the differential amplifier unit. The power supply subsystem provides following power sources: ±10 V power source for the operational amplifier and the differential amplifier, ±5 V power source for the GMR chip, 1 mA∼100 mA DC current source for the DC coil and 5 V reference voltage for the system.

In order to get the typical performance of the GMR current sensor before being processed by the thermal compensation unit and the differential amplifier unit, the terminals of the power supply of the GMR chip was connected to the ±5 V outputs of the power supply subsystem, and the voltages were directly measured from the two outputs of the GMR chip, as shown in Figure 7.

## 4. Typical Performance

The performance of the GMR current sensor, which consists of static, dynamic and thermal characteristics, determines its application. Some performance parameters of the sensors were experimentally characterized and are presented below.

### 4.1. Static Characteristics

**Figure 8.**
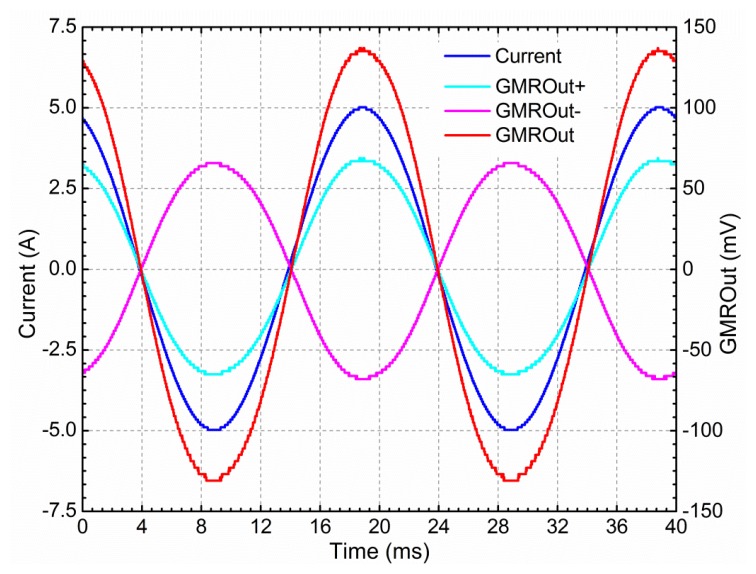
Waveforms of GMR current sensor. Current is the AC current passing through the wire, measured by the Tektronix TCP312. GMROut+ and GMROut− are outputs of the GMR chip. GMROut is the differential output of the GMR chip.

**Figure 9.**
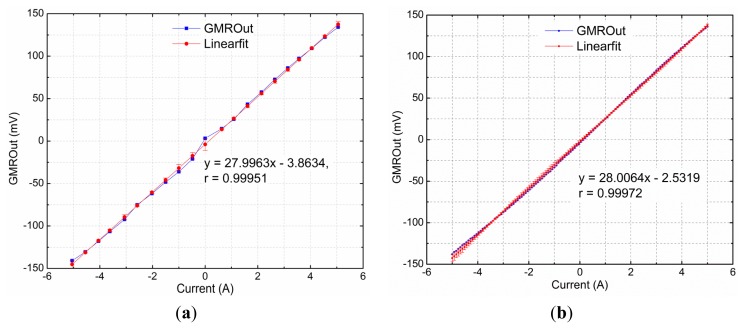
(**a**) DC linear analysis of GMR current sensor. (**b**) AC linear analysis of GMR current sensor. The output voltage offset is less than 5 mV when the measured current and the bias current of DC coil are both 0, and the value is 149 mV when the measured current and the bias current of DC coil are respective 0 A and 10 mA. GMROut is the differential output of the sensor in the corresponding measured current, and the effect of bias current of DC coil has been eliminated. Linearfit is the data of linear regression.

The static characteristics include the sensor's range, sensitivity, linearity and accuracy. The GMR current sensor has high sensitivity, as much as ten times the sensitivity of a Hall effect-based sensor [25]. As the GMR chip is supplied by a dual power supply (±5 V), the output voltage offset is less than 5 mV when the measured current and the bias current of DC coil are both 0 A, and the value is 149 mV when the measured current and the bias current of DC coil are respective 0 A and 10 mA. The effect of the DC coil was eliminated and ignored in the following performance analyses. When the current to be measured was 10 A peak to peak with frequency at 50 Hz, the environmental temperature *T* was 20 °C, the power supply voltage of the sensor *V_S_* was ±5 V, the distance from the wire to the sensor *r* was 5 cm, the length of the magnetic ring's gap *d* was 1 cm, and the angle between the magnetic field direction and the axis of sensitivity *θ* was 0°, the output waveforms of the GMR current sensor is shown in Figure 8. The linear response of the sensor is shown in Figure 9.

In the case of 0∼±5 A DC input range, the sensitivity of the GMR current sensor was 27.9963 mV·A^−1^, and the zero-current drift was 3.8634 mV, and the linearity was 0.99951. In the case of 0∼±5 A AC input range, the sensitivity of the GMR current sensor was 28.0064 mV·A^−1^, and the zero-current drift was 2.5319 mV. The linearity was 0.99972, which became higher as the measured current range decreased. Figure 9 also shows that the largest errors occurred when the measured currents were near −5 A. The measurement resolution of the GMR current sensor is 10 mA for the 0∼±5 A input range.

**Figure 10.**
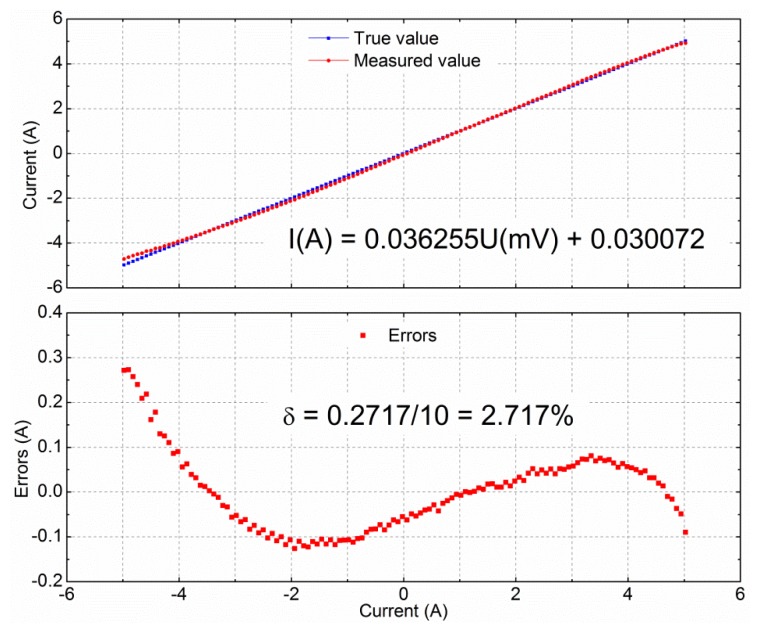
Accuracy analysis of GMR current sensor. True value is the true value of the current measured. Measured value is the calculated value of the Equation (4), according to measured value of the sensor. Errors is the deviation of the system from the true value. When the measured current range is 0∼±5 A, The function of the current is *I* = 0.036255 *U* + 0.030072, and the maximum deviation is 2.717% at −5 A.

Accuracy is one of the most important parameters when designing sensors. As shown in Figure 10, when the input AC current was below 10 A peak to peak, the input current can be obtained from the output of the GMR current sensor, using the following fitting equation:

(4) I=0.03626U+0.03007

where *I* is the measured input current in Ampere, *U* is the output of the GMR current sensor in millivolts. The maximum deviation of Equation (4) is 2.717%.

The related coefficients can be adjusted to appropriate values using the differential amplifier unit. Usually, the lower the measured current range, the higher the accuracy and the lower errors. This empirical finding is shown in Table 1. When the measured current range was 0∼±2 A, the linearity increased to 99.993%, and the maximum deviation decreased to 1.757%.

### 4.2. Dynamic Characteristics

**Figure 11.**
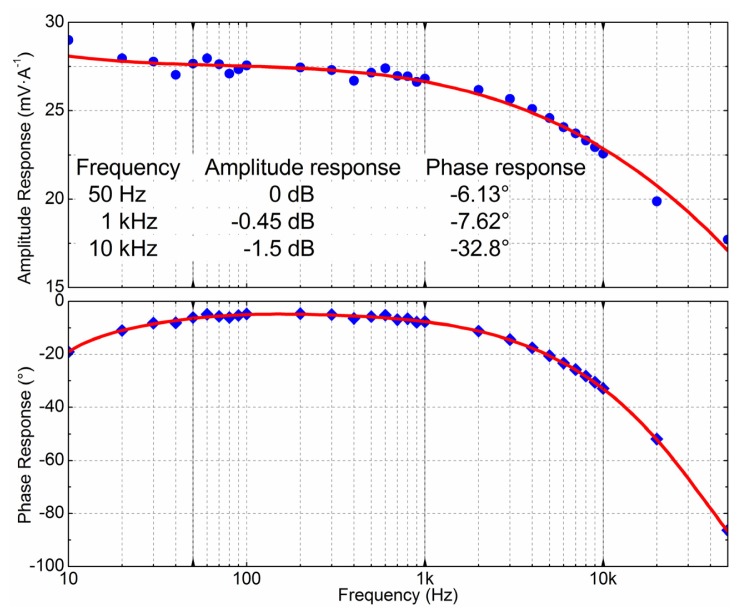
Frequency response of GMR current sensor. The way to obtain the frequency response is measuring pure sinusoidal current with different frequencies. At the reference frequency of 50 Hz, the amplitude response is 0 dB (28 mV·A^−1^), and the phase response is −6.13° lag. The corresponding data is −0.45 dB and −7.62° at 1 kHz, −1.5 dB and −32.8° at 10 kHz, respectively.

The multilayered GMR chip can sense high frequency magnetic fields up to 1 MHz. By measuring pure sinusoidal current with different frequencies, the frequency response of the sensor was obtained, which is shown in Figure 11. The GMR current sensor worked very stably from 50 Hz to 1 kHz. The amplitude response was 28 mV·A^−1^, and varied less than 5%, about −0.45 dB at the reference amplitude response of 50 Hz. The phase response lagged by approximately −6° but only varied a little. From 1 kHz to 10 kHz, the amplitude and phase response of the GMR current sensor were gradually attenuated. The amplitude response attenuation was about −1.5 dB at the reference amplitude of 50 Hz, and the phase response lagged from −6° to nearly −33°.

**Figure 12.**
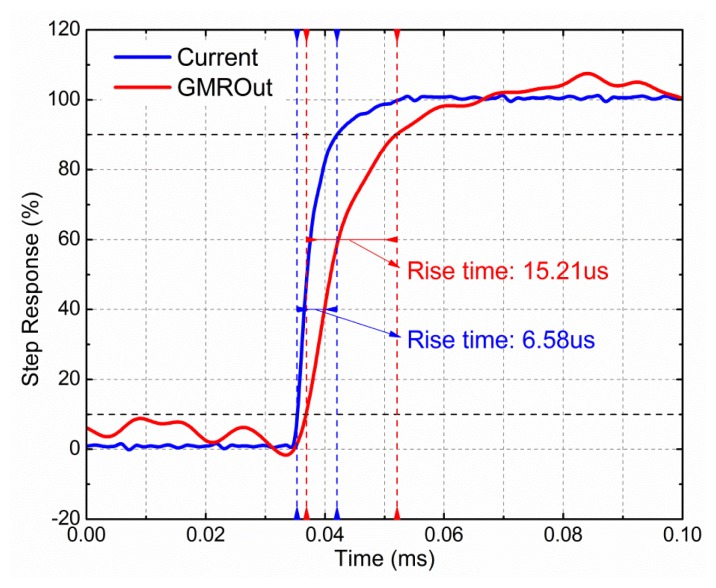
Step response of GMR current sensor. Current is the step current signal generated by the waveform generator Agilent 33250A, with the rising time of 6.58 μs. **GMROut** is the differential output of the GMR current sensor, with the rising time of 15.21 μs. Therefore, the rising time of the step response of the GMR current sensor is 8.63 μs, according to the Equation (5).

The step response of the GMR current sensor is shown in Figure 12. The step signal was generated by a waveform generator (Agilent 33250A). Measured using a Tektronix TCP312, the rise time of the step current was 6.58 μs, and the rise time of the output voltage of the GMR current sensor in response to the step current was 15.21 μs. It is recognized that an infinitely steep current step is impossible to be realized. Therefore, the rise time of the GMR current sensor is evaluated from the relation:

(5) $$T_{\text{rise}}=T_{\text{out}}-T_{\text{in}}$$

where *T_rise_* is the rise time of the step response of the GMR current sensor, *T_out_* is the rise time measured on the output of the GMR current sensor, and *T_in_* is the rise time of the applied current step. Therefore, the rise time of the step response of the GMR current sensor was about 8.63 μs.

### 4.3. Thermal Characteristics

Thermal characteristic is another important performance parameter of a sensor. The working environment of the GMR current sensor can widely fluctuate, and the applications of the sensor are directly limited by the thermal characteristics of the sensor. To characterize the thermal property of the GMR sensor, we first define the maximum change of the amplitude response in temperature as:

(6) $$\text{TMCA}=\frac{{\left(\frac{V_{O}}{I}\right)}_{\max}-{\left(\frac{V_{O}}{I}\right)}_{\min}}{{\left(\frac{V_{O}}{I}\right)}_{T\text{ref}}}\times \frac{1}{\left(T_{\max}-T_{\min}\right)}\times 100\%$$

where *TMCA* is the maximum change of the amplitude response in temperature, *V_O_*/*I* is the amplitude response of the GMR current sensor, *T* is the working temperature, “*T*_ref_” indicates the value of the reference temperature, usually 20 °C, and “max” indicates the maximum value of amplitude response, and “min” indicates the minimum value of amplitude response.

**Figure 13.**
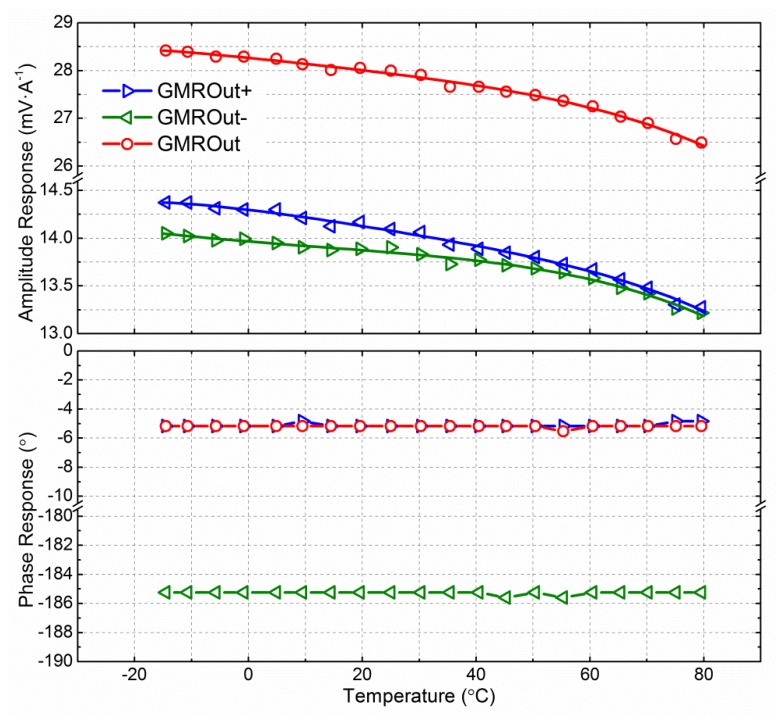
Temperature response of GMR current sensor. GMROut+ is the positive output of the GMR current sensor. GMROut- is the negative output of the GMR current sensor. GMROut is the differential output of the GMR current sensor. The amplitude response was 28.43 mV·A^−1^ at −15 °C, but decreased to 26.49 mV·A^−1^ at 80 °C. The differential output of the GMR current sensor decreased with the rising temperatures, and the *TMCA* is 0.0751%·°C^−1^. In contrast, the phase response was independent of temperature, and the phase lag with respect to the input current was −5.18°.

The thermal response of the GMR current sensor with a constant voltage power supply and 50 Hz alternating input current is shown in Figure 13. Due to limitations in material processing and manufacturing technology, the four resistive elements shown in Figure 4 are slightly asymmetrical. Therefore, the magnitude of the positive output and the magnitude of the negative output of the sensor were slightly different. According to the feature of the Wheatstone bridge, there is a phase lag of 180° between the positive output and negative output of the GMR current sensor, and the differential output has the same phase degree with the positive output of the GMR current sensor, as shown in Figure 13.

The differential output of the GMR current sensor decreased with rising temperature. The amplitude response was 28.43 mV·A^−1^ at −15 °C, but decreased to 26.49 mV·A^−1^ at 80 °C. The *TMCA* is 0.0751%·°C^−1^. In contrast, the phase response was independent of temperature, and the phase lag with respect to the input current was −5.18°, as shown in Figure 13.

In order to sustain the high degree of accuracy of the GMR current sensor, thermal compensation measures, such as a constant current power supply, should be considered. A generalized impedance converter with reference input current can be utilized to perform the thermal compensation [26,27]. This compensation technique is compatible with the Wheatstone bridge circuit, and could successfully bring *TMCA* from −0.024%·°C^−1^ to −0.007%·°C^−1^.

In the present work, a kind of simple-structure thermal compensation unit was used to compensate the thermal characteristics of the GMR current sensor. As the sensitivity and output of GMR materials are linearly related to its input power supply, a voltage power supply that increases with the rising temperature can compensate for the decreased sensitivity and output of GMR materials with the rising temperature.

**Figure 14.**
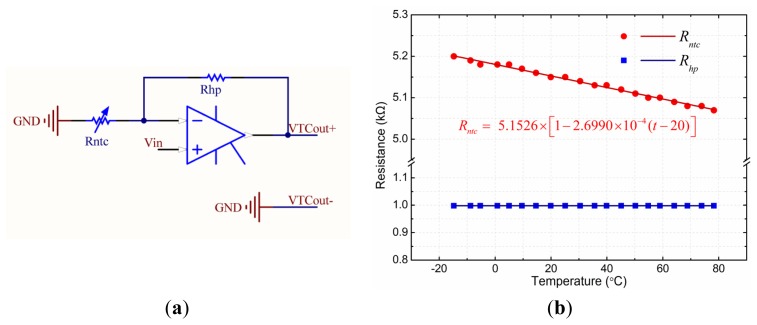
(**a**) Circuit diagram of the thermal compensation unit. (**b**) Thermal characteristics of the compensating resistors. V_in_ is the input voltage of the thermal compensation unit. VTC_out+_ and VTC_out−_ are output of the thermal compensation unit, respectively connected to the V+ and V− of the GMR chip (as shown in Figure 4). R_ntc_ is a linearized NTC thermistor. Its resistance is 5.1526 kΩ at the reference temperature of 20 °C, and the temperature coefficient of resistance (TCR) is −2.6990 × 10^−4^ °C^−1^. R_hp_ is a high precision resistor, and the resistance is 0.9980 kΩ with almost no change in temperature.

As shown in Figure 14(a), *V_in_* is the input voltage of the thermal compensation unit. *VTC_out+_* and *VTC_out-_* is the output of the thermal compensation unit, respectively, connected to the *V_+_* and *V*_−_ of the GMR chip (as shown in Figure 4). *R_ntc_* is a linearized NTC thermistor, and *R_hp_* is a high precision resistor. The output of the thermal compensation unit that is connected to the power supply of the GMR chip can be described by Equation (7):

(7) $${\text{VTC}}_{\text{out}+}=V_{\text{in}}(1+\frac{R_{hp}}{R_{\mathrm{ntc}}})$$

The thermal characteristics of *R_ntc_* and *R_hp_* are shown in Figure 14(b). The resistance of *R_ntc_* is 5.1526 kΩ at the reference temperature of 20 °C, and the temperature coefficient of resistance (TCR) is −2.6990 × 10^−4^ °C^−1^. The resistance of *R_hp_* is 0.9980 kΩ with almost no change in temperature.

**Figure 15.**
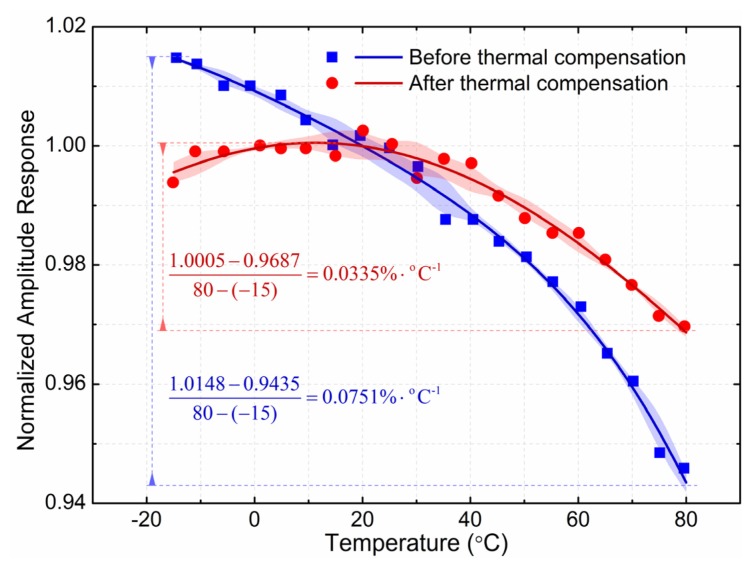
Thermal compensation of the GMR current sensor. The power supply of the GMR chip is 5 V constant voltage supply before the thermal compensation, and the V_in_ (as shown in Figure 14(a)) of the thermal compensation unit is 5 V when it is applied. The normalized amplitude response is 1.00 at the reference temperature of 20 °C. The maximum change of the amplitude response is 0.0751%·°C^−1^ before the thermal compensation, while the value reduces to 0.0335%·°C^−1^ after the thermal compensation.

With a 50 Hz alternating input current, the thermal compensation of the GMR current sensor is shown in Figure 15. The power supply of the GMR chip is 5 V constant voltage supply before the thermal compensation, and the *V_in_* (as shown in Figure 14(a)) of the thermal compensation unit is 5 V when it is applied. The normalized amplitude response is 1.00 at the reference temperature of 20 °C.

Before the thermal compensation, the amplitude response decreased monotonically with rising temperature. The maximum normalized value was 1.0148 at the temperature of −15 °C, while the minimum normalized value was 0.9435 at the temperature of 80 °C, and the *TMCA* is 0.0751%·°C^−1^ before the thermal compensation. After the thermal compensation, the amplitude response was parabolic with rising temperature. The maximum normalized value was 1.0005 at the temperature of 10 °C, while the minimum normalized value was 0.9956 and 0.9435 respectively at the temperature of −15 °C and 80 °C, and the *TMCA* decreased to 0.0335%·°C^−1^ after the thermal compensation. With this kind of simple-culture thermal compensation unit, the sensor obtained the appreciable reduction in temperature dependence.

Software compensation is another simple and effective method to achieve this purpose, in which, the current and the temperature are measured simultaneously. The output of the GMR current sensor can be calibrated and modified based on the measured temperature response curve using software compensation.

## 5. Applications in Smart Grid

**Figure 16.**
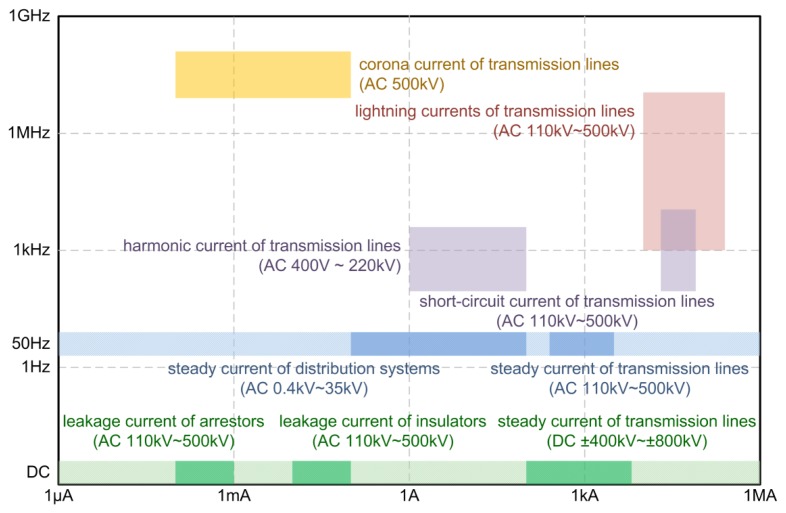
Current measurement requirements of a smart grid.

A variety of currents need to be measured in the smart grid. The coverage of the magnitude and the frequency of typical currents in the smart grid are shown in Figure 16. The magnitude of currents varies from 1 μA to 200 kA, and the frequency of currents varies from DC to 100 MHz, and the working state varies from steady-state to transient-state. Many of the currents in the figure are typical values found in the transmission lines and distributed networks. The diversity of these requirements is well matched by the versatility of GMR current sensors as described earlier.

### 5.1. Comparison of Current Sensors

Current sensors play an extremely important role in power systems for the purpose of protection and control. Presently, current sensors applied in power systems mainly include current transformers (CTs), Rogowski coils, shunts, fiber-optic current transformers (FOCTs), fluxgate sensors, Hall effect sensors and GMR effect sensors, and they are mainly based on the following physical principles: Faraday's law of induction, Ohm's law of resistance, Faraday effect and magnetic field sensors [25,28,29]. The performance and characteristics of the current sensors mentioned above are compared in Table 2.

The current transformer, with its advantages of high stability and high breakdown voltage, is the most widely applied for alternating current sensing in traditional power systems. Because it is based on Faraday's law of induction, it can't be applied in direct current measurement, and with its disadvantages of large size, high price, limited bandwidth and large consumption of metal resources, it is only used in power stations and substations. The Rogowski coil, with favorable features of high bandwidth and capability of measuring large currents, is mainly used for transient large current sensing, and can be easily installed on the transmission lines as a small size and low price unit. However, Rogowski coils have poor accuracy when sensing small and low frequency currents, which limits their broader application.

Shunts are mainly applied in power electronics and direct current converter stations. They are the most cost effective solution, but their disadvantages are obvious: the measured current has to be interrupted into the sensor, an overcurrent may permanently damage it, and the intrinsic inductance limits the accuracy and bandwidth. Fiber-optic current transformers have developed rapidly in recent years. Their advantages of effective isolation from high potentials, immunity against electromagnetic interferences, and wide bandwidth offer a wide potential application in power systems. However, the extremely complicated structure, high price, susceptibility to the temperature and polarization under direct current are problems that need to be solved.

Hall effect current sensors are mainly applied in non-contact current measurements. And indeed, currently, most non-contact current measurement probes are based on the Hall effect. However, the problems of the Hall effect sensor are low sensitivity, low breakdown voltage, and susceptibility to the temperature, which limit it to applications in high voltage power systems. The fluxgate technology can significantly improve the accuracy of magnetic field sensors such as Hall effect sensor, but because of its high cost and size requirements, fluxgate technology is usually only employed in calibration systems, diagnosis systems and laboratory equipment.

Compared with the current sensors mentioned above, the GMR effect sensor has advantages of high sensitivity, high linearity, small volume, low cost, simple structure and lower susceptibility to the temperature, which make it the most promising for current measurement in smart grids, especially in distributed transmission lines.

### 5.2. Distributed Monitoring

One major challenge in a smart grid is how to realize real-time monitoring of each node in the distributed electrical power system. Currently, real-time sensor systems such as traditional current transformers and fiber-optic current transformers, are only applied in some key places like power stations and substations, and there are currently no suitable real-time sensor systems capable of monitoring each distributed transmission line in the smart grid. With the advantages of small volume, low cost and simple-structure, the GMR current sensor can be readily implemented to monitor large-scale distributed power systems, and to provide accurate real-time information for each grid.

### 5.3. Steady-State Monitoring

In an electrical power system, the steady-state current of various electrical equipment must be monitored, such as transmission lines, power substations, AC transformer stations, DC converter stations, distribution networks, and user networks. The current can be either direct current or alternating current. In the case of DC converter stations, measurement of both direct and alternating current is necessary. The optical current transformer and fluxgate current transformer are the two most commonly-used current transformers in such DC converter stations. However, these two types of current transformers are expensive, complex, and possess adverse environmental effects. In contrast, the GMR current sensor we describe here has excellent DC and AC performance, is cost-effective, and can be readily applied to steady-state monitoring.

### 5.4. Transient-State Monitoring

In a smart grid, transient-state currents, such as lightning currents, switch impulse currents, harmonic currents and leakage currents, also needs to be monitored. However, these currents have large amplitudes and frequency ranges, making accurate measurements difficult. For instance, lightning current can reach as high as 200 kA, with frequencies as high as 100 MHz, and leakage current can be as small as 1 mA. The saturation field of multilayered GMR materials reaches 2,000 Oe (1 Oe = 0.1 mT), and a sensitivity of 0.1%·Oe^−1^. In addition, the saturation field of spin valve GMR materials reaches 50 Oe and a sensitivity of 1.0%·Oe^−1^. Therefore, we can utilize GMR current sensors based on these two kinds of materials to measure wide ranges of currents.

### 5.5. Power Monitoring

The sensitivity and output of GMR materials are linearly related to their input power supply. If we connect the power supply pins of the GMR current sensors to measure an electrical system, the output of the GMR current sensor is related to the voltage and current of the measured electrical system. Therefore, we can calculate the system's instantaneous power. A possible device used to measure alternating instantaneous power [21], and a circuit used to measure the discharge power of a battery using a GMR sensor have been developed [30]. A kind of wattmeter based on an anisotropic magnetoresistive (AMR) sensor was designed and tested experimentally [31], and a practical magnetoresistive wattmeter was designed to measure active power at industrial frequencies, which reached the 700 W power level with an uncertainty of less than 1% [32].

Taking into account the power delivered to a cosine circuit, the voltage and current of the load can be described as:

(8) $$\begin{array}{l} u(t)=\sqrt{2}V_{\text{rms}}\cos(\omega t+\theta )\\ u(t)=\sqrt{2}I_{\text{rms}}\cos(\omega t) \end{array}$$

where *V_rms_* is the root mean square of the voltage, *I_rms_* is the root mean square of the current, *ω* is the angular frequency of the circuit, and *θ* is the initial phase of the voltage.

The instantaneous power of the load can be described as:

(9) $$\begin{array}{cc} p(t) & =u(t)i(t)\\ & =\sqrt{2}V_{\text{rms}}\cos(\omega t+\theta )\cdot \sqrt{2}I_{\text{rms}}\cos(\omega t)\\ & =V_{\text{rms}}I_{\text{rms}}\cos\theta +V_{\text{rms}}I_{\text{rms}}\cos(2\omega t+\theta ) \end{array}$$

The relationship between output and input of the GMR sensor can be described as:

(10) $$\begin{array}{cc} u_{\text{out}} & =u(t)(Ai(t)+B)\\ & =\sqrt{2}V_{\text{rms}}\cos\;(\omega t+\theta )\left(A\sqrt{2}I_{\text{rms}}\cos\;(\omega t)+B\right)\\ & =2AV_{\text{rms}}I_{\text{rms}}\cos(\omega t+\theta )\cos(\omega t)+\sqrt{2}BV_{\text{rms}}\cos\;(\omega t+\theta )\\ & =AV_{\text{rms}}I_{\text{rms}}\cos\theta +AV_{\text{rms}}I_{\text{rms}}\cos(2\omega t+\theta )+\sqrt{2}BV_{\text{rms}}\cos\;(\omega t+\theta ) \end{array}$$

where *A* and *B* are the sensitivity and offset output of the GMR sensor. *AV_rms_I_rms_*cos*θ* is related to the active power of the load, *AV_rms_I_rms_*cos(2*ωt* + *θ*) is related to the reactive power of the load, and *AV_rms_I_rms_*cos*θ* + *AV_rms_I_rms_*cos(2*ωt* + *θ*) is related to the instantaneous power of the load.

To obtain the active power, Ramírez Muñoz *et al.* designed a low-pass filter to remove the AC frequency components [32]. In the works of this paper, with the aid of a digital signal processing technique, both the active power (DC component) and the reactive power (2-harmonic components) are extracted from the output signal, and then the instantaneous power of the load is calculated and the effect of the GMR sensor offset is eliminated.

**Figure 17.**
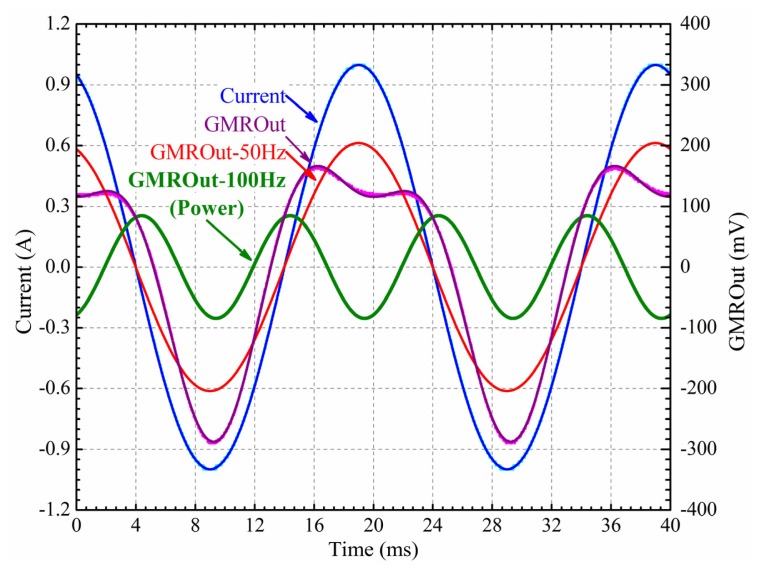
Power monitoring of GMR sensor. The 10 Ω resistor is powered by a 50 Hz and 10 V peak to peak AC voltage source. The two power supply terminals of the GMR chip are connected to the two terminals of the l0 Ω resistor load. Current is the AC current passing through the 10 Ω resistor load. GMROut is the differential output of the GMR current sensor. GMROut-50Hz is the 50 Hz frequency component of the GMROut, which is caused by the asymmetry of the Wheatstone bridge, and the frequency is the same as the voltage of the 10 Ω resistor load. GMROut-100Hz is the 100 Hz frequency component of the GMROut, which showed the instantaneous power of the 10 Ω resistor load.

To demonstrate the capability of GMR current sensor mentioned above to measure instantaneous power, the GMR current sensor was used to measure alternating instantaneous power delivered to a 10 Ω resistor. The resistor was powered by an AC voltage source at 50 Hz and 10 V peak to peak. As shown in Figure 17, the output of the GMR current sensor contained two frequency components: 50 Hz and 100 Hz. The 100 Hz frequency component showed the alternating instantaneous power of the resistor, and the 50 Hz frequency component was caused by the asymmetry of the Wheatstone bridge.

As mentioned, due to material processing and manufacturing technology limitations, it is impossible to manufacture the four resistive elements shown in Figure 4 identically. This results in voltage variations between the positive and negative outputs of the GMR current sensor when no current passes through the wire being measured. Therefore, there is a component in the output of the GMR current sensor, which the magnitude of the component is linearly related to the voltage of the load, and frequency is the same as the voltage of the load. This offset needs to be taken into account and mitigated in practice.

## 6. Conclusions

The design and development of a GMR current sensor for smart grid applications were discussed. Characterization of the sensor was done to study its static, dynamic and thermal characteristics. The static characteristics of the sensor consist of the following performance parameters: operation range, sensitivity, linearity, and accuracy. The sensitivity of the sensor is 28 mV·A^−1^. At an input range of 0∼±5 A, the linearity reached 99.972%, and the maximum deviation was 2.717%. At an input range of 0∼±2 A, the linearity reached 99.993%, and the maximum deviation was 1.757%. Furthermore, dynamic characterization of the sensor showed that the frequency response of the GMR current sensor was −0.45 dB at 1 kHz input current and −1.5 dB at 10 kHz input current. Lastly, the thermal characterization showed that the maximum change of amplitude response in temperature (*TMCA*) of the GMR sensor was 0.0751%·°C^−1^, and the value decreased to 0.0335%·°C^−1^ with a simple-structure thermal compensation unit.

The GMR current sensor described in this paper can be readily applied to monitor a smart grid in real-time. In the case of distributed monitoring, steady-state monitoring, and transient-state monitoring, the GMR current sensor provides excellent performance, and is cost-effective. Moreover, with appropriate design modifications, it can also be used for power monitoring, which potentially has a vast market in residential power systems. With its high sensitivity, high linearity, small volume, low cost and simple structure, the proposed GMR current sensor has bright prospects in the measurement and monitoring of smart grids.

## Figures and Tables

**Table 1.** Static characteristics of GMR current sensor in different measured range.

**Range**	**Linearity**	**Maximum deviation**	**Equation**
0∼±5 A	99.972%	2.717%	*I* = 0.03626*U* + 0.03007
0∼±2 A	99.993%	1.757%	*I* = 0.03626*U* + 0.00797

**Table 2.** Performance comparison of existing current sensors applied in power systems.

***Sensors***	***CT***	***Rogowski***	***Shunt***	***FOCT***	***Fluxgate***	***Hall***	***GMR***
***Volume***	large	small	small	small	large	small	small
***Price***	high	low	low	extra high	high	low	low
***Frequency Range***	0.05∼10 kHz	0.1∼100 MHz	kHz∼MHz	∼300 MHz	∼100 kHz	∼1 MHz	∼5 MHz
***DC Capability***	No	No	Yes	Yes	Yes	Yes	Yes
***Sensitivity***	1 V·A^−1^	10 mV·A^−1^	1 mV·A^−1^	high	high	10 Oe	0.01 Oe
***Dynamic Range***	1 A∼100 kA	0.1∼100 kA	mA∼kA	1 A∼3 kA	1 A∼10 kA	10 mA∼35 kA	1 mA∼10 kA
***Non-linearity***	0.05%	0.05%	0.01%	0.2%	0.001%∼0.5%	0.1∼1%	0.01∼0.05%
***Temperature Coefficient***	--	--	--	−0.4%·°C^−1^	--	−0.3%·°C^−1^	−0.1∼ −0.4%·°C^−1^
***Isolation***	complex	complex	complex	simple	complex	complex	complex
***Breakdown Voltage***	high	high	low	high	high	low	high
